# A Bibliometric Analysis of Crowdsourcing in the Field of Public Health

**DOI:** 10.3390/ijerph16203825

**Published:** 2019-10-10

**Authors:** Lingling Wang, Enjun Xia, Hao Li, Wei Wang

**Affiliations:** School of Management and Economics, Beijing Institute of Technology, Beijing 100081, China; 3120160717@bit.edu.cn (L.W.); wangwei666892@163.com (W.W.)

**Keywords:** crowdsourcing, public health, CiteSpace, bibliometrics

## Abstract

With the characteristics of low cost and open call, crowdsourcing has been widely adopted in many fields, particularly to support the use of surveys, data processing, and the monitoring of public health. The objective of the current study is to analyze the applications, hotspots, and emerging trends of crowdsourcing in the field of public health. Using CiteSpace for the visualization of scientific maps, this study explores the analysis of time-scope, countries and institutions, authors, published journals, keywords, co-references, and citation clusters. The results show that the United States is the country with the most publications regarding crowdsourcing applications for public health. Howe and Brabham are the two leading authors in this field. Further, most of the articles published in this field are found in medical and comprehensive journals. Crowdsourcing in public health is increasing and diversifying. The results of this study will enable and support the analysis of the specific role of crowdsourcing in the public health ecosystem.

## 1. Introduction

Crowdsourcing was first definitized by Jeff Howe in 2006, to represent the act of organizations outsourcing their tasks to an undefined and large group of people [1]. Brabham described crowdsourcing as an online, distributed problem-solving and production model [2]. Crowdsourcing allows access to a large pool of public volunteers, saves time in collecting data, reduces costs, and accelerates the speed of innovation [3,4]. With increasing globalization and continuing internationalization trends, the flattening effect of Globalization 3.0 has created an environment that encourages the growth of crowdsourcing [5]. Crowdsourcing can extend the innovation activities of enterprises to an infinite and vast network space—it also explores, utilizes, and integrates the innovation resources of the whole society and the wisdom of the society through the Internet [6,7,8,9]. From the fields of innovative design to hygiene, positioning services, and new product development, crowdsourcing quietly subverts business models and traditional social structures [10,11].

The Internet has greatly reduced the cost of information transfer and the boundaries of participating activities. In this context, the concept of health 2.0 has been proposed with the practice of crowdsourcing in the field of health communication [12]. Every health sector can benefit from a crowd of tasks that can facilitate research [3]. There is no doubt that crowdsourcing creates a great opportunity in health, hygiene, and medical research. As appointed by Swan, crowdsourcing health studies are the union of three contemporary tendencies, namely “citizen science,” crowdsourcing, and Medicine 2.0 [13]. Medicine 2.0 or Health 2.0 announces individuals actively participating in their health care, especially utilizing Web 2.0 technology [13]. Like Internet 2.0, Terry argues that Health 2.0 promotes self-creation, sharing, community concepts, and user self-empowerment, all of which coincide with the aim of crowdsourcing [14]. 

In recent years, the involvement of crowdsourcing in hygiene and health has been of scholarly interest. Perrine et al. summarized that different types of crowdsourcing projects can be adopted in health promotion, research, and care [3]. Maged et al. pointed out that, leveraging the power of crowdsourcing, the citizen can easily engage in public and environmental health surveillance in order to provide crowdsourced maps through social web and smartphones [15]. Brabham argued that crowdsourcing offers the opportunity to improve health actions via public involvement online [16]. Wazny conducted an overview analysis to examine how crowdsourcing has been used in global health, including diagnosis, surveillance, nutrition, public health and the environment, education, genetics, psychology, and general medicine [17]. In summary, crowdsourcing brings together the best of scientists, the public, and online communities. Further, it offers an effective approach to obtain data, track health, and monitor records, etc. Hence, crowdsourcing creates a huge opportunity for researchers to conduct research on health promotion, health monitoring, health maintenance, and behavioral surveying.

With 4.02 billion Internet users and 5.14 billion unique mobile subscribers [18], online platforms and mobile apps for crowdsourcing have been developed to improve public health [19], particularly in tracking diet and exercise, aiding smokers’ cessation, preventing binge drinking, and disseminating health communication, etc. Public health commits to improving the quality of life by preventing and treating diseases and, nowadays, many subfields of public health have drawn scholars’ attention, such as community health, behavioral health, mental health, occupational safety, gender issues in health, and sexual health [20,21,22]. Harrison C et al. used crowdsourcing to predict unreported outbreaks and foodborne illnesses based on the business review website Yelp [23]. Wilson J et al. conducted an online survey of US adults about their perceptions of chiropractors to test the feasibility of using the Amazon Mechanical Turk (MTurk) [24]. Crowdsourcing contests provide an opportunity for online citizens to participate in public engagement. For example, a competition was organized to draw public attention to heart disease by mapping the locations of automated external defibrillators [25]. Another contest experiment was that Joseph D et al. asked participants to design and upload videos to raise awareness about condom use and HIV testing in China [26,27]. In modern public health science, it may require multidisciplinary scientists to collaborate on problem-solving, data surveys, collection, and processing. Brabham described a framework for crowdsourcing adoption in public health from a four-type typology: knowledge discovery and management, distributed human intelligence tasking, broadcast search, and peer-vetted creative production [16].

**Figure 1:**
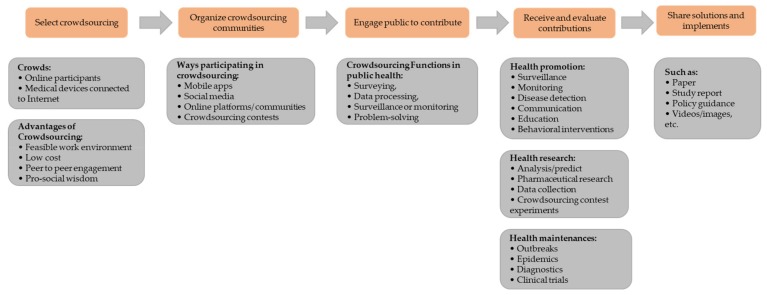
The application process of crowdsourcing in the public health contexts.

Based on the above analysis, we can illustrate the relations and the process of crowdsourcing applications for public health, as shown in Figure 1. The application of crowdsourcing in the field of public health can be divided into five processes, including selecting crowdsourcing, organizing crowdsourcing communities, engaging the public to contribute, receiving and evaluating contributions, sharing solutions and implements. Crowdsourcing can provide not only online citizens, but also scientists with a variety of information. Leveraging the feasible and low-cost characteristics of crowdsourcing, online citizens can easily participate in public health activities and share their ideas/knowledge. Meanwhile, increasing numbers of medical and hospital diagnostic devices are now connected to the Internet, which allows scientists to track and monitor the behavior of the crowd, even without letting them know. When organizing a crowdsourcing community, mobile apps, social media (Twitter, Facebook, Weibo), online platforms and crowdsourcing contests are multiple paths for researchers to consider. The Amazon MTurk, a labor market platform, is mainly used to collect data or publish tasks. Crowdsourcing contests have become an effective public health engagement tool, which has been utilized to conduct medical experiments. The real power and uniqueness of crowdsourcing adoption in public health is surveying, data processing, surveillance, and problem-solving. Specifically, crowdsourcing can contribute to three domains: health promotion, health research, and health maintenances. Finally, crowdsourcing sponsors can share solutions and applications in order to improve public health.

Most of the previous studies have only conducted experiments to explore the function of crowdsourcing in the specific field of public health, such as improving public awareness of sexual health, obtaining solutions to medical problems, and collecting medical big data, etc. Limited studies have been published related to a systematic review or overview of crowdsourcing for public health. Among them, some studies extracted data from reports via PubMed, EMBASE, and Google, but no research has utilized the Web of Science (“WOS”) database. The systematic review by Ranard et al. illustrated the scope of crowdsourcing in health and medical research, but only contained 21 studies [19]. The narrative review by Swan explained the adoption of crowdsourcing in health research studies up to 2011 [13]. Moreover, no scholars carry out bibliometric analysis on the application of crowdsourcing in public health or related topics. This study uses a bibliometric approach to analyze the adoption of crowdsourcing in the field of public health and presents research hotspots, evolution history and emerging trends. Using co-citation analysis and co-occurrence analysis, we look at the Web of Science (“WOS”) publication data related to crowdsourcing and public health from 2006 to 2019.

This study may contribute to existing research from three aspects. First, this study is the first bibliometrics analysis of crowdsourcing applications for public health using CiteSpace software. We use bibliometric analysis to provide a new insight that was not conducted comprehensively in previous studies. Second, we offer a better understanding of the emerging trends of the adoption of crowdsourcing in the field of public health, through summarizing the role of crowdsourcing in public health. Third, the analytical framework and the results concluded in this study will provide research basis and directions for future bibliometric analysis in public health ecosystem. As this study explores the general application of crowdsourcing in public health, it could serve as a sound foundation for future research that focuses on the specific roles of crowdsourcing in concrete tasks, e.g., surveying, data processing, monitoring, etc.

The rest of this study is organized as follows. Section 2 introduces the materials and methods. Section 3 conducts the time and space scope analysis of crowdsourcing research in the field of public health. Knowledge domains and emerging trends of crowdsourcing’s application in public health is displayed in Section 4. Section 5 draws the main conclusions of this study and points out future research directions and limitations.

## 2. Materials and Methods

### 2.1. Introduction to Bibliometrics

In 1955 Garfield proposed an approach for searching scientific literature with citation indexes and, since then, citation analysis has gradually become an important research method in the field of scientific metrology [28]. Pritchard suggested a proper name for this subject, bibliometrics, which combined mathematics and statistical methodology [29]. Norton applied bibliometrics as a tool to measure texts and information [30]. In recent years, bibliometric analysis has been widely utilized in the interdisciplinary research field in order to identify the development of hidden or emerging subjects [31,32,33]. From an objective and quantitative perspective, bibliometric analysis is the typical approach that uses citation relationship to generate effective material for scientists [34,35]—hence, it can reflect the hotspots, evolution, and emerging trends within a specific field [29,36,37]. 

### 2.2. Data Source and Search Strategy

We retrieved data from the core collection database of Web of Science (WOS), limiting search to the Science Citation Index (SCI), Social Science Citation Index (SSCI), Conference Proceedings Citation Index- Science (CPCI-S), and Conference Proceedings Citation Index-Social Science & Humanities (CPCI-SSH) [38,39]. This study analyzes publications from 2006 to 2019, since the “crowdsourcing” was proposed by Howe in 2006 [1]. The search rule was: TS = ((crowd$sourcing) AND (health OR hygiene* OR public near/2 health)). The search scope included existing research results of article, proceeding papers and reviews. After refinement, 308 documents were retrieved. The search was conducted in April 2019 and the summary of search details is shown in Table 1.

### 2.3. Analysis Tools

Two tools were used in the analysis, namely (A) CiteSpace V and (B) Excel 2016. (A) CiteSpace V software was used to conduct visualization and knowledge graph analysis in this study. CiteSpace V is a visual analysis tool developed by Professor Chaomei Chen (Drexel University, Dalian University of Technology, Changjiang Scholar) based on the JAVA platform, which can realize co-citation analysis, keyword co-occurrence analysis, and collaborative analysis of institutional authors, etc. [40,41]. Compared with other visualization software, CiteSpace V has the advantages of more convenient data processing, better visualization and easier interpretation. Therefore, it can meet the requirements of the literature co-citation and keyword co-occurrence analysis of large samples. (B) To perform the analysis, Microsoft Excel 2016 was used to count the annual quantities of publications for crowdsourcing research.

### 2.4. Parameter Settings of CiteSpace

CiteSpace V with version 5.2.R 2.3.26.2018 for 64-bit windows was used in this study. In the time slicing, the time span was from 2006 to 2019 and the years slice was set as 1. In the text processing, we chose all term source, including title, abstract, author keywords and keywords plus [36]. In addition, we selected pathfinder to prune the merged network because it can simplify the network and highlight the important structural features [42,43,44,45].

## 3. Time and Space Scope Analysis of Crowdsourcing Research in the Field of Public Health

### 3.1. Analysis of Publications’ Quantity

**Figure 2:**
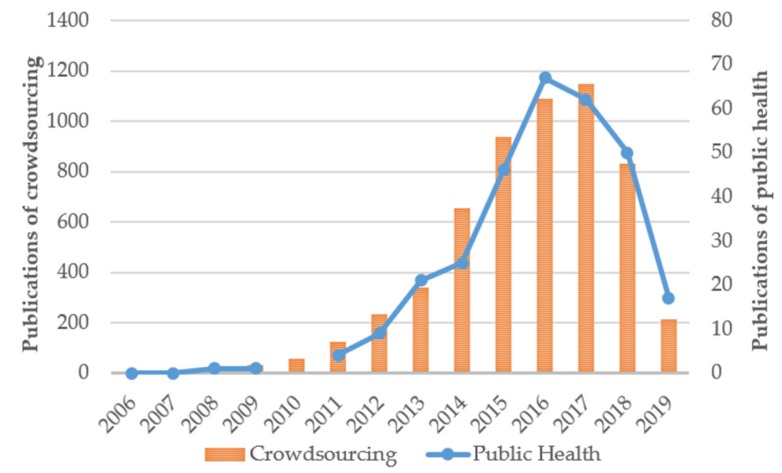
Numbers of publications related to crowdsourcing and crowdsourcing research in the field of public health in the WOS core collection, published from 2006 to 2019.

Figure 2 shows the statistical results for 5664 publications related to crowdsourcing research and 308 records for crowdsourcing applied in the field of public health in the WOS core collection, published from 2006 to 2019. Given these results, the bar chart indicates the total amount of literature on crowdsourcing research and the line chart shows the results in the field of public health. Since the concept of crowdsourcing was proposed in 2006, it was not until 2008 that the adoption of crowdsourcing in the field of public health occurred. The number of publications related to crowdsourcing saw a significant increase from 2011 to 2014—2015 to 2018 is the steady stage wherein the annual quantity exceeds 800, with the largest number in 2017. According to literature of crowdsourcing in public health, the growth trend is basically consistent with the whole crowdsourcing research; the number of papers is growing rapidly after 2014 and the largest number is 67 publications in 2016. Since we retrieved data in April 2019, it is expected that the amount of literature in the field of public health will continue to increase.

### 3.2. Analysis of Countries and Institutions

Citespace V can use annual rings to display the number of papers published by countries, cooperation and centrality. The size of the annual rings serves as the quantity of posts, and the outermost purple circle shows the centrality [41]. We select country and institution as network nodes respectively, with the data extraction object of Top 30, and the path data is visualized by pathfinder.

**Figure 3:**
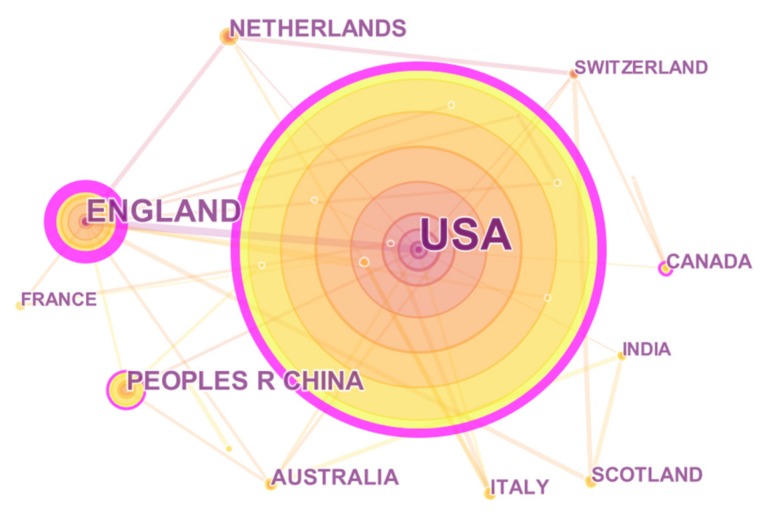
Network graph of countries/religions.

The map in Figure 3 shows that 308 publications were contributed by 24 countries/regions. The country with the most publications concerning crowdsourcing applied in public health is the United States (197), followed by England (34), China (21), and the Netherlands (13). From the perspective of betweenness centrality, the top two countries/religions were England (centrality = 0.65) and USA (centrality = 0.55), indicating that they have direct or indirect cooperation with many countries in the co-existing network, such as Canada, Netherlands and China. In recent years, researchers in China have published 21 papers. Although the research started late (the first time of publication was 2015), the number of papers has increased year by year.

As shown in Table 2, we list the top ten core research institutions that contributed to crowdsourcing research in public health. USA is the country with the largest number of academic outputs, and its main research institutions are the University of North Carolina (11), University of Pennsylvania (9), UC San Francisco (9), Columbia University (8), New York University (6), etc. Similarly, The University of Edinburgh (9) and the London School of Hygiene & Tropical Medicine (6) are the leading institutions in England/UK. Among them, UC San Francisco (centrality = 0.23), WHO (centrality = 0.12), the University of North Carolina (centrality = 0.11), and the London School of Hygiene & Tropical Medicine (centrality = 0.11) are the core institutions that drive crowdsourcing research in the field of public health.

### 3.3. Analysis of Authors and Co-Authors

**Figure 4:**
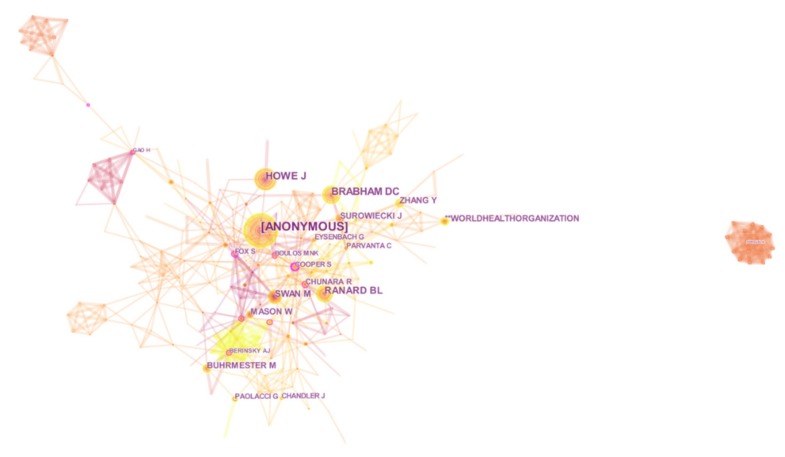
Map of co-authors collaboration network.

The author collaboration network of crowdsourcing research was described in Figure 4. The significance of Anonymous (63), Howe (40), Brabham (35), Ranard (32) and Swan (27) can be clearly appreciated. On the report of figure information, Anonymous has produced the most papers regarding to crowdsourcing applied in public health. Hence, we list the top 10 authors and co-authors with the most articles contributed by a worth of 298 authors in Table 3. The Hirsch index, or h-index, and citations have been included for each scientist. Looking at Figure 3 and Table 3, we find that Cooper (centrality = 0.21) and Gao (centrality = 0.2) are two authors with a high degree of cooperation in this field. Tucker (10), Tang (8), Zhang (7) are the top three authors with productive publications. In terms of co-citations, Mason has been cited the highest due to having the most indicators for the number of citations. However, Howe is the core co-author on crowdsourcing applications for public health.

### 3.4. Analysis of Co-Cited Journals

The table below illustrates the 11 most journals from the entire 502 of journals according to crowdsourcing applied in public health, as seen in Table 4. From the perspective of the number of publications, J MED INTERNET RES (99) and PLOS ONE (97) are the two journals with the most articles, followed by NATURE (59), J GEN INTERN MED (52), LANCET (51), and *J AM MED INFORM ASSN* (46). Many papers published on medical journals represent that crowdsourcing has been used as an advanced tool in public health research field. In addition, ‘Nature’ and ‘Science’ are comprehensive top-level journals that provide research frontiers and hotspots and build knowledge base for medical, biological, economics, management, and psychology in the field of public health research.

**Figure 5:**
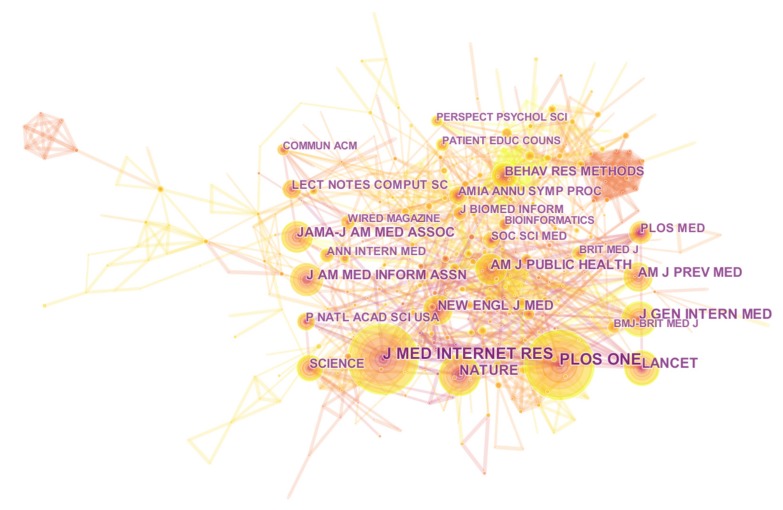
Visualization of co-citation journals analysis

When identifying core journals in a research field, the number of posts is not the only indicator, and the centrality and cited frequency are equally important. Figure 5 shows visualization of co-citation journals analysis. CLIN PSYCHOL SCI (0.13), BMC PUBLIC HEALTH (0.12) and AM J PUBLIC HEALTH (0.11) are the top three centrality journals for medical research. These journals offer academic platforms for medical researchers, which indicates that applying crowdsourcing in public health has received unanimous attention.

## 4. Knowledge Domains and Emerging Trends of Crowdsourcing’s Application in Public Health

### 4.1. Analysis of Co-Occurring Keywords

The keywords reflect the core and focus of a paper. The top 10 keywords on crowdsourcing applications in public health are listed in Table 5. When turning to the keywords, it was interesting to report that the analysis is focused on depression (centrality = 1.13), smoking (centrality = 1.01), and children (centrality = 0.91), and is measured through the Internet, social media, and online communities. Therefore, it indicates that most crowdsourcing applications connect to public health via the Internet, online platforms, etc.

### 4.2. Analysis of Co-Cited References

**Figure 6:**
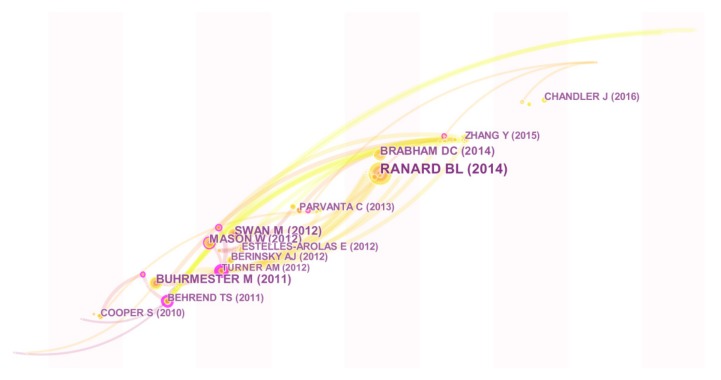
Visualization of reference time zone analysis.

Based on co-cited references, CiteSpace V can generate a research evolution graph to display the development of crowdsourcing applications in public health with a time zone version. We selected cited references as the network node and adopted the path search algorithm (pathfinder) to analyze [46]. Therefore, we can identify key research results on this topic, as seen in Figure 6. These scholars and their research results have played important roles in promoting the development of crowdsourcing research in the field of public health.

Table 6 shows Top 10 key references in crowdsourcing applications for public health. The publication “Crowdsourcing as a model for problem solving: Leveraging the collective intelligence of online communities for public good,” published in 2008 by Brabham, presents the motivation of participants to participate in the crowdsourcing activities and explore the potential of crowdsourcing applications for public sensors [2]. The most cited article is “Amazon’s Mechanical Turk: A New Source of Inexpensive, Yet High-Quality, Data?” by Buhrmester, 2011, published in the journal of the Association for Psychological Science, which describes and evaluates the potential contributions of the MTurk (a crowdsourcing platform) to psychology and other social sciences [47]. The two highly cited articles are foundation of online crowdsourcing and its applications for public health. 

The key research references can reflect the development of crowdsourcing as applied to public health., and the main findings are as follows:

In 2011, Behrend used crowdsourcing to collect survey data for behavioral research. The authors present that the application of crowdsourcing is an efficient and appropriate alternative to university participant pools [48]. This paper with a high degree of centrality has become the foundation for crowdsourcing applied in the social survey. 

In 2012, Swan pointed out that contemporary public health faces challenges, including rising costs, worsening outcomes, ‘diabesity’ epidemics, and an expected shortage of doctors. Crowdsourcing will be an effective tool to collect health big data and to realize the vision of preventive medicine by 2050 [49].

In 2014, Brabham argued that crowdsourcing has the potential to be a method for improving public health science. Crowdsourcing offers several effective ways toward improving health behaviors through participant engagement online, such as the knowledge discovery and management, the distributed human intelligence projects, the broadcast search and the peer-vetted creative production methods [16]. Meanwhile, Ranard created a taxonomy to characterize past applications of crowdsourcing in the field of health and medical research. The application of crowdsourcing can improve the quality, cost, and speed of research programs [19].

In 2015, Zhang used crowdsourcing contests to shape a “bottom-up” approach and described two creative contributory contests (CCC) to enhance sexual health campaigns [50]. This study may be useful for other groups expanding community engagement in sexual health science.

In 2016, Chandler summarized the MTurk’s (an online crowdsourcing platform) data quality with an emphasis on results related to clinical psychological research. MTurk is a fast and cost-effective approach to collect nonprobability cases that are more diverse than those commonly adopted by psychologists [51].

In 2018, Créquit mapped the diverse adoptions of crowdsourcing in the field of health to assess the health areas that are using crowdsourcing and the crowdsourcing projects used. The application of crowdsourcing is growing in health promotion, exploration, and care. However, the definition of crowdsourcing logistics and crowdsourcing participants’ characteristics is often lacking in research reports [3].

Overall, crowdsourcing has been widely used to collect data, make surveys, solve problems and monitor. Since 2012, crowdsourcing has gained important attention in the field of public health. Many scholars have begun to study the functions of crowdsourcing in personal care, biological agents, sexual health communication, etc. Furthermore, the Amazon Mturk is the most used platform, and data processing is the main type of crowdsourcing in this field. Crowdsourcing contests are an efficient and responsible way for scientists to conduct medical experiment, and to improve the awareness of public health. In addition, we find several research gaps, such as the ethical problems when applying crowdsourcing in public health, characteristics of participants in the crowdsourcing process, the effectiveness of crowdsourcing without the Internet, etc.

### 4.3. Analysis of Evolutionary Path

**Figure 7:**
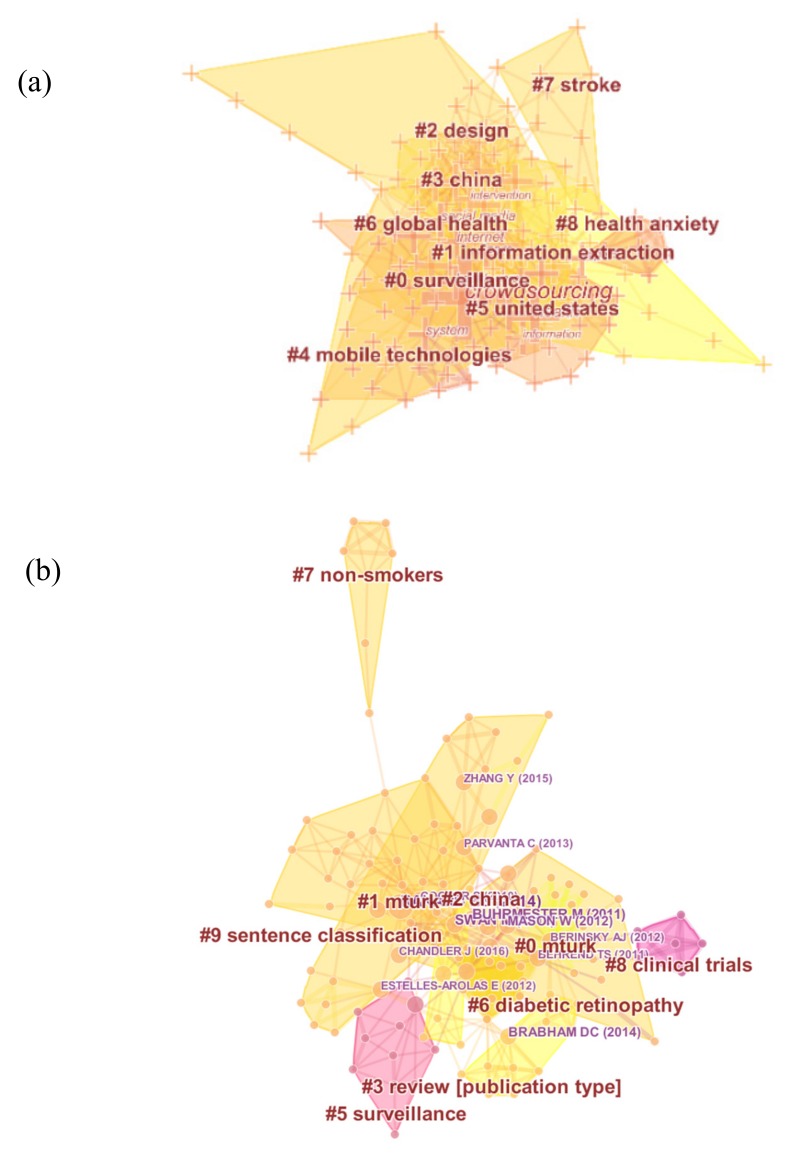
(**a**) Visualization of keywords clusters analysis; (**b**) Visualization of reference co-citation analysis.

The title words and keywords can reflect the research focus of publications. Therefore, through the co-occurrence analysis of the title words and keywords, we can explore research hotspots and frontiers in the scientific field. The clusters of keywords and references (extracting title terms) are shown in Figure 7. As we can see, keywords and references are divided into nine clusters, respectively.

In Figure 7a, the largest cluster #0 is labeled as “surveillance”, following by cluster #1 “information extraction”, cluster #2 “design”, cluster #3 “China”, cluster #4 “mobile technologies”, cluster #5 “United States”, cluster #6 “global health”, cluster #7 “stroke”, and cluster #8 “health anxiety”. In Figure 7b, there are 10 clusters from large to small: cluster #0 “mturk”, cluster #1 “mturk”, cluster #2 “China”, cluster #3 “review”, cluster #5 “surveillance”, cluster #6“diabetic”, cluster #7 “non-smokers”, cluster #8 “clinical trials”, and cluster #9“sentence classification”.

Given these results, we can summarize the publication types, application technology, research domains, and distribution. Firstly, the main type of publications is review. It means that many scholars conduct systematic review to describe the applications of crowdsourcing in public health. Next, the Amazon Mturk is the most used platform. In recent years, social media and mobile applications have become new tools to offer health advisory and collect information. Then, with the development of global health, crowdsourcing applications have spread to many regions, especially in China and the United States. Further, the potential of crowdsourcing applications for public health includes surveillance or monitoring, information extraction, design, etc. Finally, research subjects contain stroke, non-smokers, clinical trials, diabetic retinopathy, and health anxiety, however, participants’ characteristics are poorly reported.

## 5. Conclusions

From a multi-dimensional, time and dynamic perspective, we use CiteSpace V to analyze the development trends and hotspots of crowdsourcing as applied to public health. This study has shown that crowdsourcing is a relevant topic, particularly in recent years and in the field of public health. As we know, this paper is the first bibliometrics analysis of crowdsourcing applications for public health using CiteSpace software. The results in this study contribute from several aspects to our current understanding.

First, with regard to the scope of time and space, the amount of crowdsourcing literature has grown sharply, and the application of crowdsourcing has spread to many domains, especially in public health and health communication. At present, the United States occupies a leading position in the domain of crowdsourcing research for public health, followed by England and China. Scholars from the Guangdong Provincial Center for Disease Control and Prevention cooperate with American universities to apply crowdsourcing in sexual health communication and HIV prevention. They are the first research team that have made outstanding contributions to the application of crowdsourcing in the field of public health in China.

Second, according to co-authors and types of journals, Howe and Brabham are the two core authors in this field. The World Health Organization has also pointed out the effectiveness of crowdsourcing in some reports. In addition, the results of research on crowdsourcing applications for public health are mainly published in medical and comprehensive journals. Medical journals include the ‘Journal of Medical Internet Research’, ‘Lancet’, and ‘American Journal of Public Health’, etc.—comprehensive journals contain ‘Nature’, ‘Science’, and ‘PloS One’, etc.

Third, through co-citation analysis, the use of crowdsourcing in public health is increasing, particularly in preventive medicine, mental health, and personalized prevention, etc. The specific applications of crowdsourcing contain data processing, surveying, surveillance, and problem-solving. Further, the crowdsourcing model not only promotes the application of healthy big data and the construction of intelligent health platform but also pushes the online process of social medical crowdsourcing.

Finally, crowdsourcing research in this field focuses on four knowledge domains, namely crowdsourcing as a medium for Internet public health communication, the application of crowdsourcing in the field of prevention and treatment, the role of crowdsourcing in the public health care ecosystem, and applied research of crowdsourcing competitions in infectious diseases and epidemiology. In addition, there are several future research directions to discuss. The first one is the application of other databases for bibliometric analysis, such as Google Scholar, which contains citations available in sources other than the WOS. Second, the WHO emphasize the importance of crowdsourcing competitions to improving public health. Hence, future studies can evaluate the role of crowdsourcing competitions/contests in public health development. Final, future research can consider ethical concerns because personal data and diagnostic results may be shared in the process of crowdsourcing. 

Our study has some limitations. On the one hand, we did not search the gray literature to identify some unpublished studies. On the other hand, we only consider online crowdsourcing, not including research without the Internet. Therefore, we may underestimate the number of studies adopting crowdsourcing in public health.

## Figures and Tables

**Table 1:** Summary of search details.

Settings	Contents
Index	SCI-EXPANDED, SSCI, CPCI-S, CPCI-SSH
Search rule	TS = ((crowd$sourcing) AND (health OR hygiene* OR public near/2 health))
Literature types	Article, proceeding papers, review
Time span	01/2006–04/2019
Total	308

**Table 2:** Top 11 productive institutions of crowdsourcing research in public health.

Rank	Count	Institution	Country/Religion
1	11	Univ N Carolina	USA
2	9	Univ Edinburgh	UK
3	9	Univ Penn	USA
4	9	Univ Calif San Francisco	USA
5	8	Columbia Univ	USA
6	6	London Sch Hyg & Trop Med	UK
7	6	NYU	USA
8	5	Univ Oxford	UK
9	5	Univ North Carolina Project China	USA & China
10	5	WHO	Switzerland
11	5	Harvard Univ	USA

**Table 3:** Top 10 productive authors and co-authors.

Rank	Author	Count	H-Index	Co-Authors	Citations
1	TUCKER JD	10	52	HOWE J	9057
2	TANG WM	8	59	BRABHAM DC	579
3	ZHANG Y	7	130	RANARD BL	179
4	TANG SY	4	60	SWAN M	3124
5	WEI CY	4	55	** WORLD HEALTH ORGANIZATION	1919
6	MERCHANT RM	4	29	BUHRMESTER M	4219
7	WAZNY K	4	9	ZHANG Y	607
8	YU H	4	69	SUROWIECKI J	19
9	MOLLAN KR	3	7	MASON W	26169
10	BROWNSTEIN JS	3	49	CHUNARA R	609

**Table 4:** Top 11 high-profile and high-cited journals.

No.	Count	Name of Journals	IF	Country/Religion
1	99	Journal of Medical Internet Research	4.945	Canada
2	97	PLoS One	2.776	USA
3	59	Nature	43.07	England
4	52	J GEN INTERN MED	4.606	USA
5	51	Lancet	59.102	USA
6	46	J AM MED INFORM ASSN	4.292	England
7	46	JAMA-J AM MED ASSOC	51.273	USA
8	45	AM J PUBLIC HEALTH	5.381	USA
9	44	NEW ENGL J MED	70.67	USA
10	42	AM J PREV MED	4.435	USA
11	40	Science	41.037	USA

**Table 5:** Summary of top 10 keywords on the crowdsourcing applications for public health.

No.	Frequency	Keywords	Centrality	Keywords
1	149	crowdsourcing	1.13	depression
2	29	health	1.01	smoking
3	19	internet	0.95	internet
4	18	social media	0.91	children
5	15	care	0.86	prevalence
6	15	system	0.86	gender difference
7	13	health care	0.86	inventory
8	11	information	0.62	online
9	11	intervention	0.57	attitude
10	11	impact	0.56	social media

**Table 6:** Top 10 key references in crowdsourcing applications for public health.

Year	Author	Title	Frequency
2008	DC Brabham	Crowdsourcing as a model for problem solving: Leveraging the collective intelligence of online communities for public good	1104
2011	M Buhrmester	Amazon’s Mechanical Turk: A New Source of Inexpensive, Yet High-Quality, Data?	4177
2011	TS Behrend	The viability of crowdsourcing for survey research	333
2012	M Swan	Health 2050: The Realization of Personalized Medicine through Crowdsourcing, the Quantified Self, and the Participatory Biocitizen	336
2014	DC Brabham	Crowdsourcing Applications for Public Health	67
2014	BL Ranard	Crowdsourcing--harnessing the masses to advance health and medicine, a systematic review	207
2015	Y Zhang	Creative Contributory Contests to Spur Innovation in Sexual Health: 2 Cases and a Guide for Implementation	41
2016	J Chandler	Conducting Clinical Research Using Crowdsourced Convenience Samples	181
2018	P Créquit	Mapping of Crowdsourcing in Health: Systematic Review	12
